# Think globally, act locally: the role of local demographics and vaccination coverage in the dynamic response of measles infection to control

**DOI:** 10.1098/rstb.2012.0141

**Published:** 2013-08-05

**Authors:** M. J. Ferrari, B. T. Grenfell, P. M. Strebel

**Affiliations:** 1 Center for Infectious Disease Dynamics, Departments of Biology and Statistics, The Pennsylvania State University, University Park, PA 16802, USA; 2 Dept of Ecology and Evolutionary Biology, Princeton University, Princeton, NJ 08544, USA; 3 Department of Immunization, Expanded Programme on Immunization, Vaccines and Biologicals, 20 Avenue Appia, World Health Organization, Geneva 1202, Switzerland

**Keywords:** measles, elimination, vaccination, mean age at infection

## Abstract

The global reduction of the burden of morbidity and mortality owing to measles has been a major triumph of public health. However, the continued persistence of measles infection probably not only reflects local variation in progress towards vaccination target goals, but may also reflect local variation in dynamic processes of transmission, susceptible replenishment through births and stochastic local extinction. Dynamic models predict that vaccination should increase the mean age of infection and increase inter-annual variability in incidence. Through a comparative approach, we assess national-level patterns in the mean age of infection and measles persistence. We find that while the classic predictions do hold in general, the impact of vaccination on the age distribution of cases and stochastic fadeout are mediated by local birth rate. Thus, broad-scale vaccine coverage goals are unlikely to have the same impact on the interruption of measles transmission in all demographic settings. Indeed, these results suggest that the achievement of further measles reduction or elimination goals is likely to require programmatic and vaccine coverage goals that are tailored to local demographic conditions.

## 1. Introduction

Following on the successes of smallpox eradication, measles has been suggested as a probable candidate for eradication [1–3]. While the feasibility of measles eradication has been debated [4], major efforts have turned towards the interim goal of reducing the burden of incidence and mortality [5,6]. In 2000, the United Nations General Assembly adopted a resolution to reduce extreme poverty by half by 2015 through a series of Millennium Development Goals. Millennium Development Goal 4 (MDG4) calls for the reduction of childhood deaths (less than 5 years) by two-thirds from 1990 to 2015. Given the high rate of childhood mortality owing to measles infection in low-income countries, the proportion of children vaccinated against measles was adopted as one of three indicators used to measure progress towards MDG4. In 2010, the World Health Assembly endorsed accelerated measles control targets for 2015; specifically, more than or equal to 90 per cent coverage with the first dose of measles containing vaccine and more than or equal to 80 per cent vaccine coverage in every district.

Despite broad improvement towards the vaccine coverage goals over the past decades, endemic measles transmission persists in much of the world and incidence has been observed to increase in recent years in sub-Saharan Africa and western Europe [7–9]. The continued persistence of measles infection probably reflects local variation in progress towards vaccination target goals, but may also reflect local variation in dynamic processes of transmission, susceptible replenishment through births, and stochastic local extinction. Identifying the degree to which local conditions mediate the performance of programmatic goals is critical to projecting the consequences of current targets and planning for the definitive goal of eradication.

By the nature of its relatively simple epidemiology—high transmissibility, lifelong immunity and no environmental or animal reservoir—measles has been the classic model infection for the development of dynamic models for studying the impact of vaccination [10–12]. These theoretical models predict shifts in dynamics of measles epidemiology as a consequence of control efforts; in particular: epidemic duration [10], a shift in the mean age of infection [10–14] and the increasing role of stochastic fadeout as a consequence of increased vaccination [15,16].

The reduction of incidence as a consequence of vaccination is expected to reduce the overall force of infection and increase the mean age of infection [10–13]. This general phenomenon has been best documented for rubella, where increasing mean age of infection increases the likelihood of congenital rubella syndrome [17]. In the case of measles, mortality risk due to infection is much higher in infants [18]; thus increasing mean age is expected to reduce childhood mortality. Shifts in the age distribution of cases may have further, unexpected consequences on transmission as both mobility and mixing may be age-specific [19].

The reduction in effective susceptible birth rate as a consequence of measles vaccination is expected to lead to shifts in the cyclical outbreak dynamics of measles [20]. Ferrari *et al.* [21] showed that in settings with strong seasonal forcing, such as that resulting from rural–urban agricultural migration [22,23], reduction of the effective birth rate of susceptibles could lead to high amplitude, multi-annual cycles. As a consequence of the deterministic shift in the amplitude of measles cycles, local transmission of measles is likely to fade out as the predicted incidence between peaks gets progressively lower [15,21]. During these periods of local fadeout natural infection is removed as a source of immunity and the susceptible population grows at a rate, equal to the birthrate times the fraction un-immunized, which is necessarily greater than or equal to the growth rate in the presence of natural infection. Thus, the increased volatility of local transmission dynamics can result in a more rapid growth of the susceptible population, over a multi-year period, for a given level of vaccination coverage.

Though the predictions of dynamic models and the response to vaccination control have been well studied for many well-documented case studies [21,24–32], the generality of the theoretical predictions has rarely been assessed due to lack of consistent, large-scale surveillance data. Here, we take a comparative approach to study the dynamical response of measles infection to vaccination using contemporary surveillance data on age-specific incidence and temporal variability of measles infection at the national scale. By comparing patterns in countries across the globe, which vary in measles vaccination coverage and demographic characteristics, we identify both general patterns of the response of measles infection to vaccination and the degree to which those general patterns are mediated by local demography.

## 2. Methods

### (a) Age at infection

The mean age of infection is expected to increase with vaccination coverage due to the reduction in average incidence and concomitant reduction in the force of infection [10,33]. We calculated the expected mean age of infection as a function of vaccination coverage using a simple, deterministic Susceptible–Infected–Recovered compartmental model (see the electronic supplementary material A for details). We assumed that a fraction *p* of children at age *A*_v_ = 1 year were immunized (i.e. vaccinated and sero-converted). We further assumed that the population was at constant size with a constant rate of mortality (i.e. type II). Thus, populations with a low mean lifespan were assumed to also have correspondingly high birth rate. While this demographic model is a poor representation of high-income countries, where the mortality rate is unlikely to be constant across ages, it is likely to be a reasonable representation of demographic processes in low-income countries that represent the bulk of current measles transmission [5]. For the purposes of illustration, we consider only the example of a single dose of vaccination. Much of the world offers a second dose opportunity, either through a routine second dose or through periodic supplemental immunization activities (SIAs). We note, however, that direct evaluation of the increase in first-dose coverage due to SIAs and second dose opportunities is difficult, as high reported coverage may be strongly biased towards those vaccinated through a routine first dose [34]. Thus, we present results here with respect to reported routine first-dose coverage as an index of overall vaccination programme performance.

To assess global patterns in the age distribution of measles cases, we analysed 77 010 records from 72 countries reported to the World Health Organization (WHO) case-based surveillance system between 2002 and 2010 (for list of countries, see the electronic supplementary material, table S3). We analysed only cases that were laboratory or clinically confirmed, or epidemiologically linked to a confirmed case. The ages of cases were reported in years and we aggregated cases across all reported years to calculate the mean age of infection for each country. The mean age of infection tended to increase from 2002 to 2010, thus the aggregated data reflect an average over that time period (see the electronic supplementary material B, figure S1).

Measles vaccination coverage is reported annually to WHO by all member states through the WHO/UNICEF Joint Reporting Form [35]. WHO derived coverage estimates for the first routine dose of measles containing vaccine (MCV1) from reported coverage data and survey results by use of computational logic [36].

We assessed the relationship between the mean age of measles infection and the MCV1 coverage and birth rate (births/population size in 2010) using a linear mixed-effects model with random intercepts for WHO global burden of disease (GBD) regions (Americas, Asia Pacific, East Asia, Eastern Europe/Central Asia, North Africa/Middle East, South Asia, Southeast Asia, Sub-Saharan Africa, Western-Central Europe) implemented with the package lme4 in the R software package [37]. We restricted this analysis to the 50 countries reporting more than or equal to 100 cases in the age-specific dataset to limit biases due to low sample size.

### (b) Measles persistence

To assess global patterns in the variability of measles incidence and measles fadeout at the country level, we analysed monthly records of measles cases reported to the WHO from 144 countries (11 in the Americas, six in Asia Pacific, one in East Asia, 16 in Eastern Europe/Central Asia, 18 in North Africa/Middle East, five in South Asia, 13 in Southeast Asia, 42 in Sub-Saharan Africa, 29 in Western-Central Europe). The number of months reported ranged from 1 to 84 months, with a median of 25.5 months (see the electronic supplementary material, table S3, for regional summaries). The years included in the dataset were 2002 to 2010, with the bulk of countries reporting in the latter years (note that a report of 0 cases is counted as a reported month).

Classically, the occurrence of a local extinction event, or ‘fadeout’, has been defined as a month for which 0 cases are reported [26,38,39] at the scale of municipalities. Aggregated data, at the country scale, are more difficult to interpret as asynchrony in epidemic fadeout at the local scale can be obscured when data are summed across municipalities [40]. In the WHO monthly surveillance dataset, only 11 of 144 countries reported months with 0 cases. As such, we adopted an alternate definition of fadeout as a month with less than 10 reported cases. While not a true extinction at the country scale, this level would indicate that local extinction is occurring in a significant proportion of municipalities, or is likely, due to demographic stochasticity. The general patterns in the results (below) are not sensitive to the choice of cutoff (i.e. defining a fadeout as less than 2–15 cases; we note that at the country-level, reports of true fade-outs (i.e. 0 cases) were rare).

To study the relationship between measles fadeout and population size, birth rate and vaccination coverage, we fit the proportion of fadeouts in the reported monthly time series using a generalized linear mixed model with binomial errors and a random intercept for WHO GBD region using the package lme4 in the R software package [37]. We note that the probability of observing a fadeout (regardless of the cutoff definition used) is expected to increase in smaller populations assuming that reporting is less than perfect; i.e. the binomial probability of observing fewer than *x* cases out of *I* true cases, for any binomial reporting rate, is a decreasing function of *I*. To verify that the observed relationships are significantly different from those we would expect by random chance we performed a bootstrap analysis of the observed data assuming different levels of binomial reporting (see the electronic supplementary material E).

## 3. Results

### (a) Age at infection

**Figure 1.**
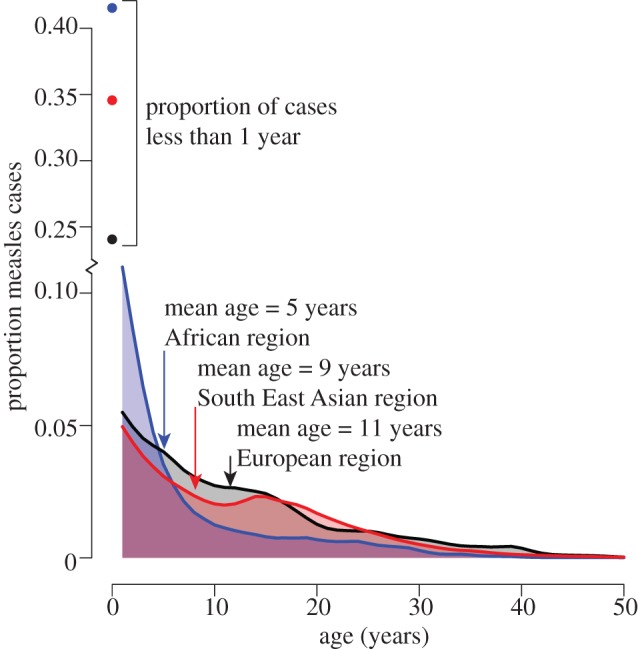
Age distribution of measles cases reported by world region (blue = African region, red = South East Asian region, black = European region) from WHO case-based surveillance system, 2002–2010.

Though classically thought of as a childhood infection (i.e. in the pre-vaccination era), the observed age distribution of measles infection worldwide has a strongly skewed distribution (figure 1). In the African and South East Asian regions, more than 41 per cent and 34 per cent of cases are observed in children less than 1 year of age. This may reflect infections in children that are too young to vaccinate (the recommended age for the first dose varies from 9 to 12 months) or children who had maternal immunity at the time of vaccination rather than a failure of vaccination programmes, *per se*. In the African region, the age distribution of cases declines rapidly: mean age of infection 5.6 years. In the Southeast Asian region, incidence declines rapidly up to approximately 10 years (mean age of infection 9.1 years), but the distribution has a long tail, with 28 per cent of cases in more than 15 years. In the European region, 24 per cent of cases fall below 1 year of age and incidence in older individuals declines less rapidly than in Africa and Southeast Asia; which is consistent with an overall lower force of infection.

**Figure 2.**
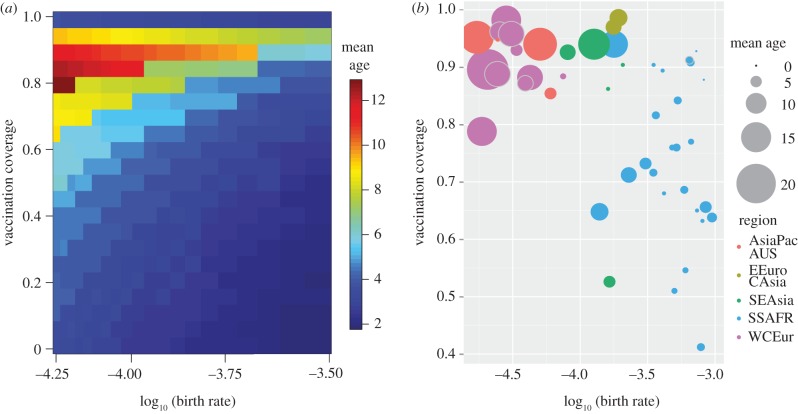
Mean age of measles infection as a function of vaccination coverage and demographic rates. (*a*) Analytical predictions from an age-structured SIR (Susceptible, Infectious, Recovered) model with a single dose of vaccine administered at 1 year of age (*y*-axis). The model assumes constant population size; longer expected lifespan implies low birth rate and low population turnover (*x*-axis). Colours and contours indicate the expected mean age at infection. (*b*) Observed mean age at infection for 50 countries with more than 100 cases reported between 2001 and 2010. The *x*-axis indicates the logarithm of the annualized birth rate (births/population size) in 2010. The *y*-axis indicates the MCV1 coverage in 2010. The size of circles indicates the mean age of measles cases. Colours indicate countries in GBD regions: Americas, Asia Pacific, East Asia, Eastern Europe/Central Asia (EEuro/CAsia), North Africa/Middle East (NAfrica/MEast), South Asia, Southeast Asia (SE Asia), sub-Saharan Africa (SS Africa), Western-Central Europe (WC Europe). Grey borders on some circles added to highlight overlapping points.

At the scale of individual countries, theory predicts that the mean age of infection should increase with vaccination coverage [10,13,33]. The rate of that increase in mean age is mediated by the demographic rates in the population (figure 2*a*); populations with high birth rates, and thus skewed age distributions, are predicted to experience a much slower change in the mean age of infection as vaccination coverage increases. Note, that at very high coverage, the mean age of infection is predicted to decline as the individuals remaining unvaccinated are disproportionately those that are younger than the age at first vaccination.

In general, countries in the European and Southeast Asian regions had higher MCV1 coverage and correspondingly higher mean age of measles cases (figure 2*b*). Countries in the African region had lower mean age of measles cases and only a very weak correlation with vaccination coverage, as predicted by the analytic model (figure 2). In a linear mixed effects model using all country data for which there were more than 100 reported cases, the negative relationship between the mean age of cases and birth rate was statistically significant (*p* < 0.00­1). MCV1 coverage was not significantly correlated with mean age of cases (*p* = 0.64). Thus, while MCV1 coverage is positively correlated with mean age of cases across all countries (linear regression: *p* = 0.049, *R*^2^ = 0.08), that relationship is not significant within regions. We note that these results are not changed if we use MCV1 coverage in 2001 or the mean MCV1 coverage from 2002 to 2010 as a covariate (see the electronic supplementary material C, table S1).

### (b) Measles persistence

Increased vaccination coverage is expected to reduce the likelihood of contact between infected and unvaccinated susceptible individuals, resulting in a higher probability of stochastic fadeout of infection. There was a weak relationship between the vaccinated proportion (MCV1 coverage) and the proportion of months with less than 10 reported cases (*p* = 0.061). Countries that had conducted any SIAs did have a marginally higher incidence of fadeout (*p* = 0.049), though there was no significant effect of the number or reported coverage of SIAs.

**Figure 3.**
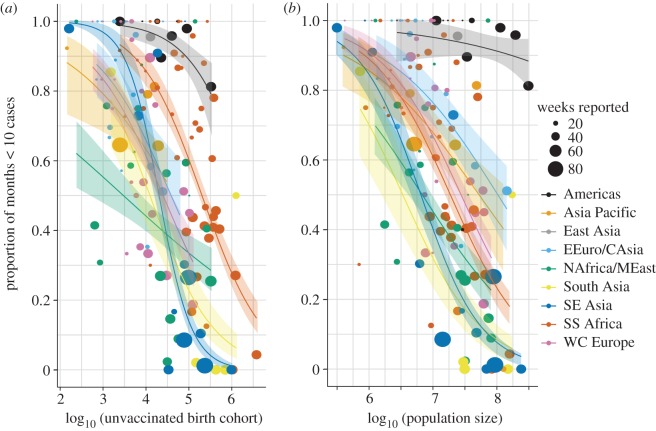
Proportion of months with less than 10 reported measles cases from the WHO monthly incidence dataset as a function of (*a*) the size of the unvaccinated birth cohort in 2010 and (*b*) the population size in 2010. Each circle reflects one country, colours indicate GBD regions. The size of each circle reflects the number of weeks reported in the dataset. Lines indicate fits of a logistic regression model for each region. Shaded regions indicate 95% confidence regions.

There is a strong negative relationship between the proportions of months with less than 10 reported cases and the size of the unvaccinated birth cohort (annual births × (1−MCV1 coverage)) (*p* < 0.001). This relationship is consistently negative across all WHO regions (figure 3*a*). The proportion of months with less than 10 reported cases scales negatively with country population size, but the effect of the unvaccinated birth cohort remains significant (*p* = 0.0018) in a model including population size (figure 3*b*, electronic supplementary material D, table S2). The slope of this relationship was greater than would be expected by random chance due to binomial sampling (see the electronic supplementary material E). This implies that the dynamics of measles fadeout are determined more by the absolute number of susceptibles than by the proportion of the population that is susceptible.

## 4. Discussion

The broad-scale coverage goals for measles vaccination, such as those adopted by the World Health Assembly in response to the Millennium Development Goals, have resulted in significant reductions in the burden of measles disease and childhood mortality [5,41,42]. The overall reduction of measles incidence is predicted to lead to shifts in the underlying dynamics of measles transmission and persistence that may impact the broader goal of eradication. The same classical models that have guided much of current vaccination policy (i.e. the 95% herd immunity threshold and the critical community size; [10]) also predict that vaccination should affect the age distribution of measles cases [10–14], should increase the rate of stochastic local fadeout [43] and increase the annual variability in incidence at the local scale [21]. Here, we illustrate, using aggregate national data, that these classic predictons do appear to hold, in general, but are mediated by local variation in birth rate. Thus, the achievement of measles reduction or elimination goals at the local (national or lower) scale is likely to require programmatic and vaccine coverage goals that are tailored to local demographic conditions.

Simons *et al.* [5] recently showed that mortality due to measles had decreased by 74 per cent from 2000 to 2010; this reduction was achieved both due to a reduction in overall incidence and to an increase in the age distribution of cases from the high-risk under-5 years age class. Here, we have shown that the mean age of measles infection is broadly correlated with overall first-dose measles vaccination coverage, which would predict further reductions in the overall case-fatality rate with increased vaccination. However, the rate at which the mean age at infection increases with vaccination coverage is negatively correlated with birth rate. Thus, in many low-income countries, where birth rates are high, we may predict to continue to see measles infection concentrated in young children who are at high risk for mortality.

Herd immunity is a central concept to the proposition that eradication is logistically feasible in the absence of perfect immunization at 100 per cent coverage [44]. As incidence declines, however, the increasing likelihood of stochastic local fadeout can obscure the evaluation of herd immunity as the absence of local transmission does not necessarily imply sufficient population level immunity. A single dose of measles vaccine administered at 9–12 months is only expected to achieve immunity in 84 per cent of individuals [45], thus, a single dose alone is not able to achieve the classic 95 per cent immunity threshold. Here, we find that there is no empirical support for a threshold first-dose vaccination coverage level at which measles is likely to fade out at the national-level; neither was there a significant correlation between first-dose coverage and the rate of measles fade out. Some of the variation in observed measles fade out may be due to differences in the administration of both first and second dose opportunities through SIAs, though the effect was weak. Though one would expect a clearer relationship between vaccination and fade out, this may reflect the challenges in using reported vaccination coverage as a measure of population immunity.

The proportion of stochastic fadeout (months with less than 10 cases) is strongly negatively correlated with both population size and the size of the unvaccinated birth cohort; i.e. the number rather than the proportion of new susceptibles in the population. Lloyd-Smith *et al.* [46] found that local persistence in wildlife populations failed to show a threshold with population size, but did increase gradually with demographic turnover rates. The results here suggest that local persistence of measles correlates with both population size and demographic rates. This suggests that broad-scale vaccine coverage goals are unlikely to have the same impact on the interruption of measles transmission in all demographic settings. Indeed, these results suggest that in order to achieve increased rates of local stochastic extinction of measles, target vaccine coverage should be scaled positively with either population size or the size of the birth cohort.

The rate of local stochastic fadeout is likely to feed back on the mean age of measles infection. In times of local measles fadeout, natural infection is removed as a source of immunity and those individuals missed by immunization programmes (due to access or refusal) will age until the next reintroduction of measles. Thus, in areas of high rates of local fadeout, we would predict to see an increase in mean age of infection relative to the predictions due to vaccination alone. Several examples of resurgent measles outbreaks in areas following periods of local elimination (Sao Paulo in 1996 [47], Burkina Faso in 2009 [9], Malawi in 2010 [8], France 2012 [7]) have been characterized by broad age distributions which are likely to result from the interaction of the age-shift due to vaccination and the ageing of the remaining susceptible population during times of local stochastic fadeout. This interaction between births and local fade out may further explain the stronger effect of birth rate than vaccination coverage on mean age of measles infection (figure 2).

Here, we have presented an evaluation of national-scale patterns as a function of both vaccine programme performance, as measured by first-dose measles coverage, and demographic rates using a broad, comparative analysis. The combined impact of vaccination and demography on susceptible recruitment has a powerful impact on measles dynamics, which underlines the key role of herd immunity in limiting epidemics [48]. Comparative analyses across a range of other pathogens underline this point [49,50].

Aggregate patterns of measles age distribution and persistence at the national scale are likely to obscure the complexities of local, sub-national transmission dynamics and vaccination coverage (due to access or refusal). Further, additional local variation in human movement [51], school attendance [52] and seasonal migration [22] are likely to affect both the age at infection and the local persistence of measles infection. Covariation of these heterogeneities with local clustering of vaccination rates and refusal is a critical area for future work as measles vaccination continues to ramp up. The relevance of these local-scale drivers reinforces, rather than detracts, from the overall conclusion of this comparative analysis.

### (a) Measles modelling and vaccination policy

In general, the development and evaluation of measles control and elimination strategies has relied on two main sources of information—country experience and modelling. Modelling can be used to generalize the lessons from individual country settings for the evaluation of novel policies. McLean & Anderson [11] used dynamic models to illustrate that optimal vaccine targeting depends on the local demographic rates and that a single age-target for routine immunization was always preferential to a two-stage introduction strategy. Since then, models have been used to evaluate the impact of the introduction of and timing of a second dose of measles vaccine [53], the effect of periodic SIAs [54] and the efficacy of outbreak response vaccination [55,56]—all leading to revisions in global measles policy [57]. Detailed case studies have been useful in validating the predictions of theoretical models such as the role of seasonality and birth rate in producing locally erratic outbreak dynamics and the maintenance of regional persistence through metapopulation dynamics [21,23]. Finally, models and surveillance data have been explicitly combined to develop policies that are grounded in both general theoretical understanding and local dynamics. Gay *et al.* [58] combined serological surveillance with model predictions to quantify outbreak risk in the UK and recommend enhanced vaccination. Where permitted by local logistics, such synthesis of serological estimation of populations at risk with models provide an especially powerful tool for targeting measles control. Where serological data are not available, cases surveillance can serve as a proxy; Simons *et al.* [59] developed models that include national surveillance data in global evaluation of progress towards the global measles mortality reduction targets.

The dynamics of measles infection and persistence, particularly as incidence declines due to the success of control measures, will be mediated by locally specific demographic forces. Our analysis here indicates that there is broad-scale correspondence between the theoretical predictions and the patterns observed across all countries. We note, however, that the individual variation from the expected relationship may reflect either local deviation from the model assumptions—e.g. due to population growth, the role of migration, local vaccine programme performance, vaccine refusal. In practice, identifying these variations from theoretical predictions could be used to identify regions that exceed programmatic expectations or to bolster elimination efforts in regions that are falling behind. Thus, while broad-scale programmatic goals have been highly successful in attaining goals for the proportional reduction of incidence and mortality, achieving the definitive goal of eradication will probably require the integration of surveillance data and quantitative models to develop control measures that are custom-tailored to local conditions.
